# Self-regulation of visual word form area activation with real-time fMRI neurofeedback

**DOI:** 10.1038/s41598-023-35932-9

**Published:** 2023-06-06

**Authors:** Amelie Haugg, Nada Frei, Milena Menghini, Felizia Stutz, Sara Steinegger, Martina Röthlisberger, Silvia Brem

**Affiliations:** 1 Department of Child and Adolescent Psychiatry and Psychotherapy, Psychiatric University Hospital Zurich, University of Zurich, Zurich, Switzerland; 2 Neuroscience Center Zurich, University of Zurich and ETH Zurich, Zurich, Switzerland

**Keywords:** Cognitive neuroscience, Reading

## Abstract

The Visual Word Form Area (VWFA) is a key region of the brain’s reading network and its activation has been shown to be strongly associated with reading skills. Here, for the first time, we investigated whether voluntary regulation of VWFA activation is feasible using real-time fMRI neurofeedback. 40 adults with typical reading skills were instructed to either upregulate (UP group, N = 20) or downregulate (DOWN group, N = 20) their own VWFA activation during six neurofeedback training runs. The VWFA target region was individually defined based on a functional localizer task. Before and after training, also regulation runs without feedback (“no-feedback runs”) were performed. When comparing the two groups, we found stronger activation across the reading network for the UP than the DOWN group. Further, activation in the VWFA was significantly stronger in the UP group than the DOWN group. Crucially, we observed a significant interaction of group and time (pre, post) for the no-feedback runs: The two groups did not differ significantly in their VWFA activation before neurofeedback training, but the UP group showed significantly stronger activation than the DOWN group after neurofeedback training. Our results indicate that upregulation of VWFA activation is feasible and that, once learned, successful upregulation can even be performed in the absence of feedback. These results are a crucial first step toward the development of a potential therapeutic support to improve reading skills in individuals with reading impairments.

## Introduction

Reading is a key skill in our society and substantially influences academic and socio-economic development^1^. As a consequence, individuals with poor reading skills, such as individuals with developmental dyslexia, suffer from disadvantages in their education and employment^2,3^, and demonstrate lower scores in overall quality of life^4,5^. In the brain, reading skills have been associated with the functionality of a diverse set of brain regions such as the inferior frontal gyrus (IFG), the intraparietal lobule (IPL), the superior temporal gyrus (STG), the left precentral gyrus (PCG), and the ventral occipitotemporal cortex (vOTC)^6,7^ which together form the brain’s reading network^8^.

One key structure of the reading network responsible for the processing of written stimuli is the Visual Word Form System along the fusiform gyrus in the ventral occipito-temporal cortex, with the Visual Word Form Area (VWFA) located in the left mid-fusiform gyrus as its core. The VWFA is suggested to process print on hierarchical posterior-to-anterior and lateral-to-medial axes, with more recent studies suggesting a subdivision of VWFA functions^9–12^. More posterior regions of the VWFA thus receive bottom-up information from the visual cortex and are particularly responsive to the presentation of orthographic stimuli and perceptual aspects of print. Anterior portions of the VWFA integrate top-down information from higher-order areas of the reading and language network and are related to lexical processing^10,11^.

Importantly, VWFA activation levels have been shown to be associated with reading skills^13,14^. Both poor-reading and illiterate individuals were found to show lower VWFA activation than typical readers^15,16^. Furthermore, also lesion studies in adults support the strong interrelation between reading performance and VWFA function^17,18^. This finding makes the VWFA a promising target for interventions aiming at improving orthographic processing. Importantly, Hirshorn and colleagues also demonstrated causality between VWFA activation and reading performance^19^. They showed that disruption of VWFA activation with intracranial electrodes led to an impaired perception of words and letters. In accordance, a normalization of VWFA signals through brain-based interventions might also support reading.

One method which allows for a fast and long-lasting regulation of brain signals in order to normalize dysfunctional neural signals is real-time fMRI neurofeedback (rtfMRI NF). rtfMRI NF enables its user to voluntarily control their own brain signals within or between predefined regions of interest (ROIs) by providing feedback on ongoing brain measures acquired with an MRI scanner^20–22^. Using this feedback information, the user can adapt their own mental processes (e.g. mental strategies) to direct the acquired brain signals in the desired direction. In the past, rtfMRI NF has been applied to a wide range of different target ROIs, including subcortical regions such as the amygdala^23–25^ or the ventral tegmental area^26–28^, and cortical regions such as the auditory cortex^29^, supplementary motor area^30^, or the visual cortex^31^. Importantly, Pereira and colleagues already demonstrated that successful self-regulation of activation within the bilateral fusiform face area, which lies in a close location to the VWFA in the left hemisphere, is indeed feasible^32^. Further, rtfMRI NF has been demonstrated to successfully improve behavioral measures linked to the targeted ROIs, such as attention^33^, memory^34^, motivation^35^, and visual perception^31^. Importantly, the normalization of dysfunctional measures has also been shown to lead to significant improvements in clinical measures for patients suffering from disorders such as depression^36,37^, post-traumatic stress disorder^38^, or Huntington’s disease^39^.

To date, however, rtfMRI NF has never been used in the context of reading and it is unknown whether activation in the VWFA can be regulated. The ability to regulate activation in this region is an important prerequisite for an application in the field of dyslexia. Therefore, we conducted a first study to investigate the feasibility of up- and downregulation of the VWFA in healthy individuals. In addition, we investigated whether, once learned, self-regulation of the VWFA would also be possible to perform in the absence of feedback.

## Methods

### Participants

45 right-handed adults were recruited for the study. They were randomly assigned to either an upregulation (UP) or downregulation (DOWN) group. Inclusion criteria included typical reading performance (reading fluency scores higher than the 30th percentile for both words and pseudowords) and non-verbal IQ scores above a threshold of 85, which resulted in the exclusion of four participants due to low performance in reading fluency. Further exclusion criteria were psychiatric or neurological disorders according to DSM-5^40^ and MRI-incompatibility (e.g. metal implants, pacemakers, claustrophobia, pregnancy). One additional participant was excluded due to an incidental finding, leaving a total of 20 participants in the UP group and 20 participants in the DOWN group. A detailed description of demographic, cognitive, and reading scores for the UP and DOWN group is provided in Table 1 in the results section. All participants provided written informed consent and were compensated 60 CHF for their participation. The study was approved by the ethics committee of the Kanton of Zurich. All methods and analyses were performed in accordance with the guidelines and regulations of the ethics committee of the Kanton of Zurich and the Declaration of Helsinki.

**Table 1:** Overview of demographic and behavioral measures in the UP and DOWN participant groups.

	UP group: mean, std	DOWN group: mean, std	Group difference
Age (in years)	26.76, 5.144	25.54, 3.60	t(38) = 0.87, p = 0.39
Sex	8 m, 12f.	10 m, 10f.	
School years	11.63, 1.3	12.2, 1.47	t(37) = 1.28, p = 0.21
Non-verbal intelligence (RIAS)	109.75, 5.58	110.68, 3.72	t(33) = 0.59, p = 0.56
Working memory (WAIS)	20.30, 4.21	19.00, 2.70	t(38) = 1.16, p = 0.25
Handedness (EHI)	78.18, 15.51	83.33, 17.56	t(37) = 0.34, p = 0.34
Mental imagery (VVIQ)	35.42, 10.17	37.5, 13.61	t(37) = 0.54, p = 0.59
Motivation	8.65, 1.23	7.95, 1.85	t(38) = 1.41, p = 0.17
Tiredness before training	3.70, 1.84	3.00, 1.03	t(38) = 1.49, p = 0.15
Mean attention during training	6.74, 1.88	6.80, 1.21	t(38) = 0.12, p = 0.91
Reading comprehension (LGVT)	57.45, 24.44	61.55, 30.10	t(38) = 0.47, p = 0.64
Reading speed (LGVT)	49.65, 25.41	55.10, 31.38	t(38) = 0.60, p = 0.55
Reading accuracy (LGVT)	71.35, 23.91	74.45, 26.97	t(38) = 0.39, p = 0.70
Reading fluency words (SLRT-II)	64.16, 18.39	72.33, 12.44	t(34) = 1.59, p = 0.12
Reading fluency pseudowords (SLRT-II)	69.81, 16.32	75.85, 15.91	t(34) = 1.12, p = 0.27
Reading fluency overall (SLRT-II)	66.98, 14.78	74.09, 11.53	t(34) = 1.62, p = 0.11

Non-verbal intelligence, handedness, and reading measures are standardized values. Motivation, tiredness, and attention were rated on a scale from 1 to 10.

### Screening and behavioral testing

Prior to study inclusion, all volunteers were screened via phone call. Participants who fulfilled the basic inclusion criteria were then provided with a set of questionnaires which they filled out online at home using RedCap (https://www.project-redcap.org/). These questionnaires included the Edinburgh Handedness Inventory (EHI) to assess the level of handedness^41^, the Adult Reading History Questionnaire^42^, the Vividness of Visual Imagery Questionnaire to get information on each participant’s abilities to perform vivid mental imagery (VVIQ)^43^, and a custom questionnaire on language skills and basic demographic information.

In addition, participants performed several cognitive tests online during a 20 min video call session with an investigator. These tests assessed working memory using the forward and backward digit span test from the Wechsler Adult Intelligence Scale (WAIS-IV)^44,45^, and non-verbal intelligence using two subtests of the German version of the Reynolds Intellectual Assessment Scale (RIAS, subtests *Odd-Item Out* and *What’s missing?)*^46^. Five participants reported being already familiar with the RIAS and were excluded from analyses concerning non-verbal intelligence.

Finally, participants underwent two reading tests in person at the MR Center of the Psychiatric University Hospital Zurich. Here, participants first performed a standardized reading fluency test (SLRT-II)^47^ where words and pseudowords had to be read out loud as fast and as accurately as possible within one minute. Then, participants underwent a standardized silent reading assessment of a longer text (LGVT)^48^ to measure reading comprehension, reading accuracy, and reading speed. In specific, participants were instructed to read as much of the text as possible within a time window of 6 min and to pick the correct answer from single-choice word options embedded in the text. The two reading tests took approximately 10 min and were repeated a second time after the MR session. Two versions of LGVT, which were randomly assigned to the participants, were used before and after the MR session to control for memory effects. Four participants reported being already familiar with the SLRT-II and were excluded from analyses involving reading fluency scores.

### Setup of the MR session

**Figure 1:**
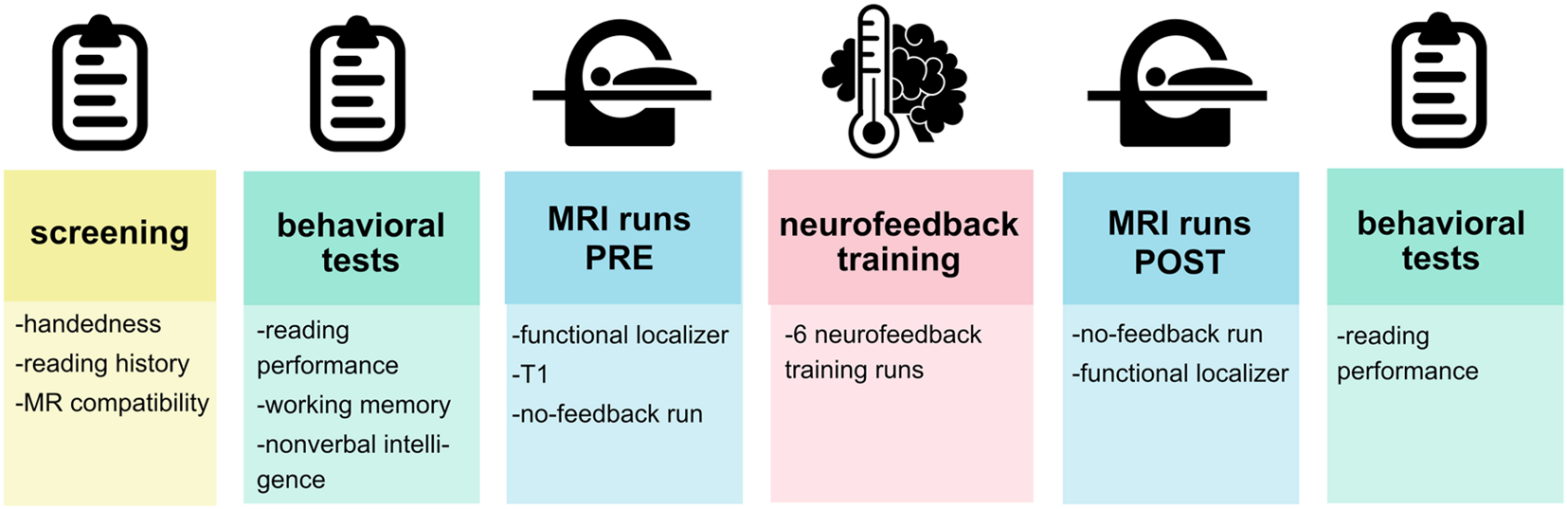
Experimental design. After an initial screening, participants underwent a set of behavioral tests on cognition and reading skills. During the MR session, participants first underwent a functional VWFA localizer, a T1-weighted anatomical scan, and a no-feedback run. Then, 6 runs of neurofeedback training were performed, followed by a break outside the scanner. After the break, participants repeated the no-feedback and functional localizer run in the MR scanner and the reading performance tests outside the scanner.

After behavioral testing, participants received instructions on the MR session. The MR session lasted approximately 1.5 h, including a break outside the MR scanner after around one hour. The session included a functional VWFA localizer and a no-feedback run each before and after rtFMRI NF training as well as six rtfMRI NF training runs. An overview of the whole experimental design including the MR session is given in Fig. 1.

### Functional VWFA localizer runs

At the beginning of each MR session, a functional localizer scan was performed to identify the individual location of the VWFA for each participant and to assess the VWFA's responsivity to words. The localizer run consisted of nine blocks of word presentation interleaved with nine blocks of checkerboard presentation. Each word block lasted 16.5 s and contained 15 German words which were presented for 800 ms each with an inter-stimulus interval of 300 ms. All words consisted of 5–8 graphemes, 2–3 syllables, and were nouns with a word frequency between seven words per million and 10,000 words per million as assessed by WordGen^49^. During the checkerboard blocks, checkerboard images were presented instead of words. To assess potential rtfMRI NF-driven changes in VWFA responsivity to word stimuli, the functional localizer was repeated a second time after NF training.

### Neurofeedback runs

**Figure 2:**
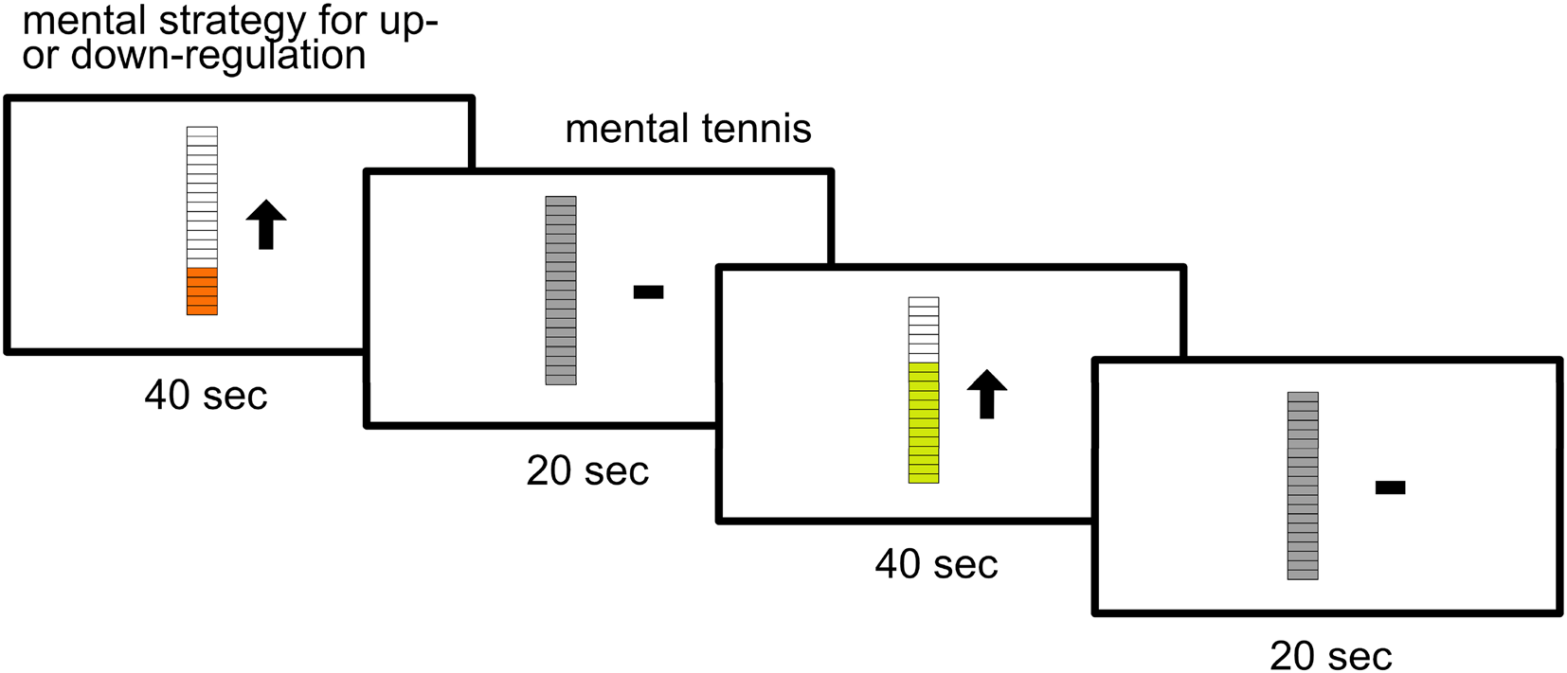
Neurofeedback paradigm. During neurofeedback runs, participants were instructed to either upregulate (UP group) or downregulate (DOWN group) their visual word form area activation using mental strategies. To prevent them from thinking about reading-related strategies during other times, participants were instructed to mentally play tennis during baseline blocks that were interleaved with the regulation blocks.

Each participant underwent a total of six NF training runs. One NF run consisted of four 20-s baseline blocks and four 40-s regulation blocks (see Fig. 2). During baseline blocks, participants were instructed to mentally play tennis. This was done to ensure that participants would be engaged in a task unrelated to reading. During the regulation blocks, participants were asked to either upregulate (UP group) or downregulate (DOWN group) their VWFA activation using mental imagery. All participants were informed about the role of the VWFA and its engagement in the reading process. Consequently, participants in the upregulation group performed reading-related mental imagery, and participants in the downregulation group performed mental imagery unrelated to reading. After each run, participants were asked about their exact mental strategy for the respective run via intercom. Further, they were asked to estimate their neurofeedback regulation performance and their subjective attention during the run on a scale from 1 (very poor) to 10 (excellent), also via intercom. Finally, participants were given time after each run to think about possible strategies for the next run. This avoided mental strategy planning during baseline periods.

Participants received continuous visual feedback on their regulation performance in form of a thermometer icon which was filled in based on ongoing VWFA activation levels. In addition, the thermometer icon changed color from red to yellow to green for successful upregulation in the UP group or downregulation in the DOWN group. To emphasize the direction of the regulation task, an upwards (UP group) or downwards (DOWN group) pointing arrow was displayed next to the thermometer icon. During baseline blocks, the arrow was replaced by a neutral line and the thermometer icon turned grey and was static.

### No-feedback runs

Before and after NF runs no-feedback runs were performed. During no-feedback runs, participants had to perform the same mental imagery as during the NF runs, but no feedback was provided. Similarly to the NF paradigm, participants had to perform four 20-s baseline blocks with mental imagery of tennis and four 40-s regulation blocks. Instead of the color-changing thermometer icon, a blue filled thermometer icon was presented during the regulation blocks. Finally, as it was done after each NF run, participants also had to estimate their regulation performance and report their mental strategy and attention after the no-feedback runs. Participants were asked to not perform a new mental strategy in the no-feedback run after NF training, but optimally use the mental strategy which worked best for them during neurofeedback training.

### Image acquisition

The MR session was conducted using a 3 Tesla Philipps Achieva MRI Scanner (Philipps, Best, The Netherlands) located at the MR Center of the Psychiatric University Hospital Zurich. All images were acquired with a 32-channel head coil. The functional VWFA localizer runs each consisted of 247 volumes using a gradient-echo T2*-weighted planar imaging (EPI) sequence with the following parameters: repetition time (TR) = 1250 ms, echo time (TE) = 35 ms, flip angle (FA) = 80°, field of view (FoV) = 197 × 197 × 138 mm^3^, 42 slices acquired in ascending order, slice gap of 0.3 mm, multiband factor 2, and voxel size = 3 × 3 × 3 mm^3^. The first 5 images were discarded as dummy scans. The no-feedback and NF runs were conducted using the same sequence with the following scan parameters: TR = 2000 ms, TE = 35 ms , FA = 82°, FoV = 220 × 220 × 110 mm^3^, 27 slices in ascending order, 1 mm slice gap, no multiband, voxel size of 2 × 2 x 3mm^3^. 130 volumes were acquired per no-feedback/NF run, preceded by an additional five dummy scans which were discarded before data analysis. Finally, a T1-weighted anatomical image was acquired using a FoV of 270 × 255 × 176 mm^3^, voxel size of 1 × 1 × 1 mm^3^, 176 slices, and 9° FA. Extensive descriptions of all scanning parameters can be found in the exam card files uploaded to our Open Science Framework project (https://osf.io/4qhdb/).

### Neurofeedback target ROI

The target ROI for NF training was defined as the 50% most active voxels during a participant’s functional VWFA localizer within a predefined VWFA mask (see Figure S1 in the Supplemental Material). This predefined VWFA mask was created based on ten spheres of varying radii around coordinates taken from the literature (see Supplemental Material Sect. 1 for a detailed description of the literature coordinates and the creation of the mask) which were chosen to cover a large part of the area associated with the VWFA (the exact mask can be downloaded from our Open Science Framework project: https://osf.io/4qhdb/). For each NF session, the predefined mask was transformed from Montreal Neurological Institute (MNI) space into the participant’s native space. Then, to determine the 50% most activated voxels, the functional VWFA localizer run was analyzed on-site using SPM12 and MATLAB2020b as soon as its image collection was finished. Preprocessing included realignment, coregistration to a single echo planar imaging (EPI) image acquired with the neurofeedback MR sequence right after the localizer run, and smoothing with 6 mm full width at half maximum. Normalization was not performed as NF training was performed in native space and because each participant’s target ROI was defined individually. Then, a general linear model (GLM) was calculated with regressors for words, checkerboards, and the six motion parameters. Based on the GLM, a contrast of interest for words versus checkerboards was computed. Finally, this contrast was then thresholded to only receive the 50% most active voxels within the literature-based VWFA mask and subsequently binarized.

### Online analysis of neurofeedback data

Online analysis of the NF runs was performed using the toolbox OpenNFT^50^ based on MATLAB and Python. OpenNFT received volumes acquired by the MR scanner in real-time using the DRIN export system implemented by Philipps. MR data were preprocessed using OpenNFT’s default preprocessing pipeline including real-time realignment and spatial smoothing. In addition, OpenNFT’s default denoising options were chosen which included the following steps: Drift removal using a cumulative GLM, Kalman filtering for spike removal, and adjustment for serial correlations using a first-order autoregressive model AR(1). Finally, OpenNFT’s default dynamic scaling approach ensured that participants would not reach ceiling or floor effects due to larger changes in signal intensity. After dynamic scaling, the signal was converted to a number between 1 (low brain activation) and 20 (high brain activation) and fed back to the participant in form of color changes of the thermometer icon.

### Offline analysis of neurofeedback and no-feedback runs

All offline analyses were performed using SPM12 (Statistical parametric mapping, http://www.fil.ion.ucl.ac.uk/spm/software/spm12/) and MATLAB 2020b 

(https://www.mathworks.com/products/matlab.html). Offline fMRI data analyses of neurofeedback and no-feedback runs included standard preprocessing steps (slice-time correction, realignment, coregistration, normalization to Montreal Neurological Imaging (MNI) space, and smoothing with 6 mm full width at half maximum) for whole brain analyses and preprocessing in native space without normalization and coregistration to the mean functional image instead of the anatomical image for ROI analyses. For both whole brain and ROI analyses, we created a GLM with regressors for regulation and baseline and the six motion regressors. In addition, motion censoring was performed to account for excessive motion (framewise displacement values above 0.9) in single images^51^.

For whole brain analyses, the contrast of interest “regulation versus baseline” of the normalized data was used as input data for the second-level GLM. Second-level GLMs were calculated for each group separately across all neurofeedback runs and for a group comparison across all neurofeedback runs. An initial threshold of p = 0.001 was used for single groups and comparisons between the two groups. Significant clusters were defined based on family-wise error corrections with p < 0.05.

For ROI analyses, the contrast of interest “regulation versus baseline” was defined for the non-normalized data. Then, mean contrast images from the VWFA ROI targeted during NF were extracted for each participant. The mean contrast values were analyzed using mixed ANOVAs with factors “run number” (pre and post, or neurofeedback run number) and “group” (UP, DOWN), and t-tests where applicable. To account for differences in initial VWFA responsiveness, the mean baseline VWFA activation during the functional localizer was added as a covariate.

### Offline analysis of functional VWFA localizer runs

Functional VWFA localizer runs were preprocessed using realignment, coregistration, normalization to MNI space, and smoothing with 6 mm full width at half maximum. An additional preprocessing analysis was performed without normalization to allow for extracting activation levels from the exact ROI that was trained during NF training. For both preprocessed datasets a GLM was calculated using regressors for words, checkerboards, and the six motion parameters. In addition, motion censoring was performed for single volumes with too much motion (framewise displacement values above 0.9). To assess the localization success, whole brain and ROI analyses were performed on a single subject level. The whole brain analysis and ROI analysis were both based on the same “words versus checkerboards” contrast. To assess activation in the right hemisphere, the literature-based mask was flipped from the left to the right hemisphere and contrast values were extracted from this contralateral right hemispheric mask. Paired t-tests using the left and right literature-based masks were performed to compare the right and left hemispheres. Further, a mixed model ANOVA with factor “run” (PRE, POST) and “group” (UP, DOWN) was used to analyze the extracted mean contrast values of the left mask. Finally, for each group, a second-level analysis with an initial threshold of 0.001 and family-wise error cluster-level correction was performed.

### Statistical analysis of behavioral data

Behavioral data were analyzed using paired and unpaired t-tests, Pearson correlations, and mixed model ANOVAs. Bonferroni correction was performed where applicable. When sphericity was not given for the ANOVAs, corrections were made using Greenhouse–Geisser. All behavioral analyses were performed with IBM SPSS 24 (https://www.ibm.com/de-de/products/spss-statistics) and MATLAB 2020b (https://www.mathworks.com/products/matlab.html).

## Results

### Behavioral and demographic measures before neurofeedback training

The UP and DOWN group did not differ significantly in their IQ scores (t(33) = 0.59, p = 0.56), working memory (t(38) = 1.16, p = 0.25), handedness scores (t(37) = 0.34, p = 0.34), or years of school education (t(37) = 1.28, p = 0.21). Importantly, the two groups also did not show any significant differences in reading measures as assessed by the SLRT-II and LGVT tests before neurofeedback training. A detailed description of all behavioral measures in the two groups, including several subtests of the two reading tests, is shown in Table 1. Finally, both groups reported similar levels of tiredness (t(38) = 1.49, p = 0.15), similar scores of motivation to participate in the neurofeedback experiment (t(38) = 1.30, p = 0.20), and did not differ significantly in their mental imagery or working memory scores.

### Functional localization of the VWFA

The functional VWFA localizer task successfully engaged and localized the VWFA in all participants but one who was in the DOWN group. That one participant demonstrated a negative value when extracting the mean beta value for the “words versus checkerboards” contrast and was, therefore, excluded from further analyses regarding NF. This was necessary because a successful localization of the NF target region in this person could not be achieved. The other participants all showed a highly significant VWFA cluster on a single-subject level (FWE-corrected p < 0.001) and positive beta values when extracting the mean beta value for the “words versus checkerboards” contrast from a VWFA mask based on literature (see Methods section and Supplemental Material Sect. 1 for a description of the literature-based mask). The UP and DOWN group did not differ significantly in their VWFA activation during the localizer task (t(38) = 1.00, p = 0.32). Further, each participant demonstrated stronger VWFA activation in their left than in their right hemisphere. The difference between left and right VWFA activation was highly significant (t(39) = 11.68, p < 0.001). An overview of all brain regions engaged during the functional VWFA localizer task before NF in both the UP and DOWN group can be found in the Supplemental Material (Figures S1 and S2, Tables S2 and S3).

### Whole-brain activation during neurofeedback training

**Figure 3:**
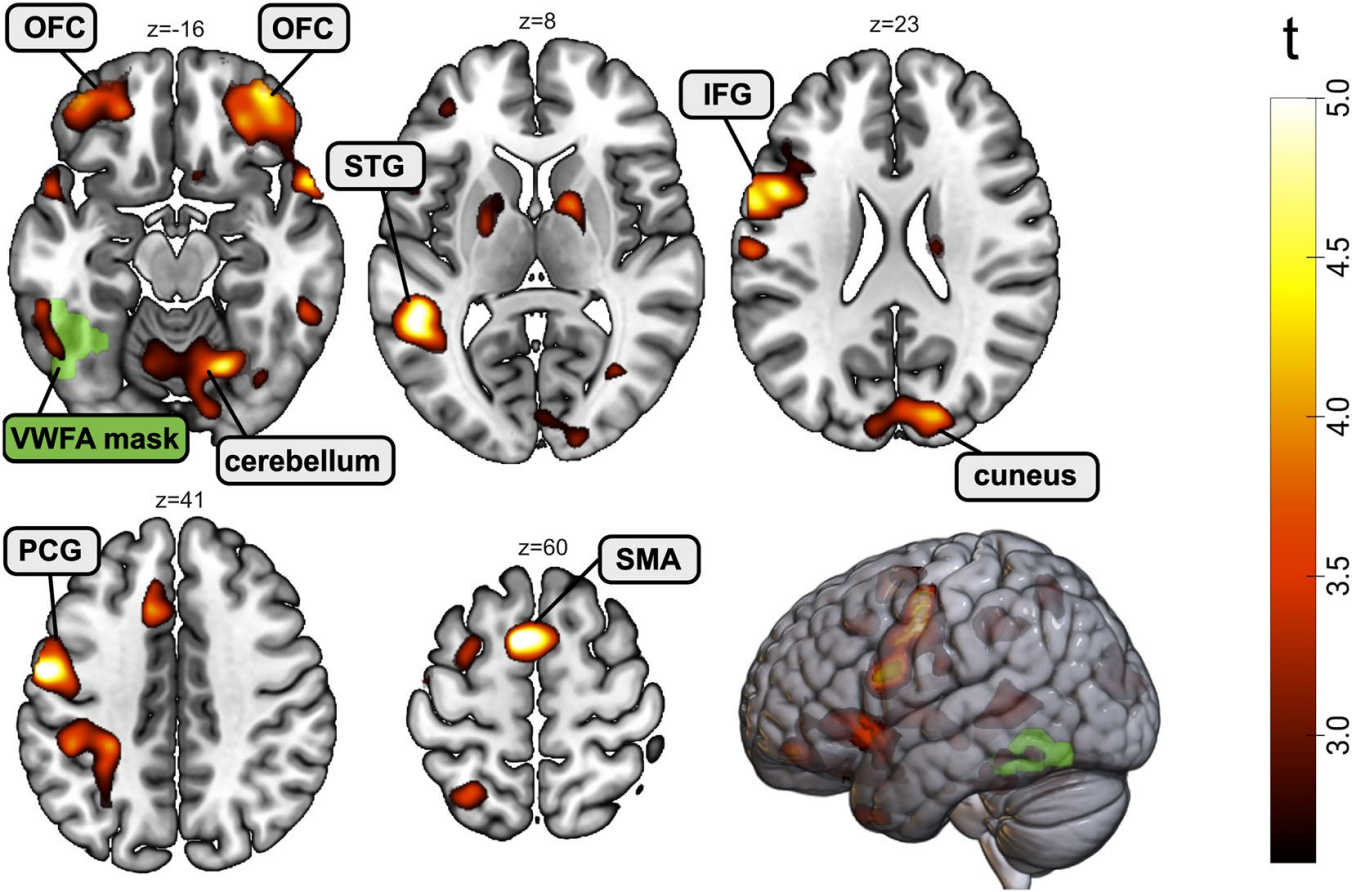
Whole brain activation during neurofeedback runs in the UP group as compared to the DOWN group. When comparing the regulation versus baseline contrast between the two groups, we observed significant (FWE-corrected p < 0.05) clusters in the left superior temporal gyrus (STG), the left inferior frontal gyrus (IFG), the left precentral gyrus (PCG), the cerebellum, orbitofrontal cortex (OFC), cuneus, and the supplementary motor area (SMA). The literature-based mask of the VWFA is depicted in green.

On a whole brain level, when contrasting the UP to the DOWN group we found significant (FWE-corrected p < 0.05) clusters across the reading network including the left precentral gyrus (PCG), the left IFG, and the left STG. In addition, we observed significant activation within the cuneus, the supplementary motor area (SMA), the cerebellum, and the orbitofrontal cortex (OFC) (see Fig. 3 and Table 2). A detailed description of the results for the UP and DOWN group individually is given in the supplementary material (Tables S4 and S5). Results for analyses using a more lenient initial threshold of 0.005 (FWE-corrected p < 0.05) additionally included the VWFA and the left IPL (see Table S6, Figure S8 in the Supplemental Material). One participant of the DOWN group consistently moved their head in one direction, which, over time, resulted in the VWFA moving partly outside the field of view. Therefore, this participant was excluded from further analysis regarding NF.

**Table 2:** Overview of clusters demonstrating higher activation in the UP than in the DOWN group for the regulation versus baseline contrast of neurofeedback runs (see Table S6 in the Supplemental Material for the results with a more lenient initial threshold of 0.005)**.**

Cluster		Peak		Coordinates			anatomy
p(FWE-corr)	equivk	p(FWE-corr)	T	x	y	z {mm}	
0.001	661	0.001	6.97	24	-62	-24	cerebellum
		0.939	3.77	42	-62	-30	
		0.999	3.35	36	-56	-32	
0.032	326	0.007	6.25	-8	6	60	supplementary motor area
< 0.001	1286	0.026	5.71	-56	-6	40	left precentral gyrus
		0.065	5.35	-48	-8	52	
		0.176	4.93	-56	2	34	
0.002	610	0.062	5.37	-42	28	-4	left inferior frontal gyrus
		0.386	4.55	-52	10	-6	
0.014	407	0.072	5.31	-52	-46	8	left superior temporal gyrus
0.001	763	0.226	4.81	36	52	-14	orbitofrontal cortex
		0.339	4.61	54	14	-18	
		0.692	4.17	26	18	-24	
0.041	303	0.586	4.29	18	-86	24	cuneus

### Activation in the visual word form area during neurofeedback training

**Figure 4:**
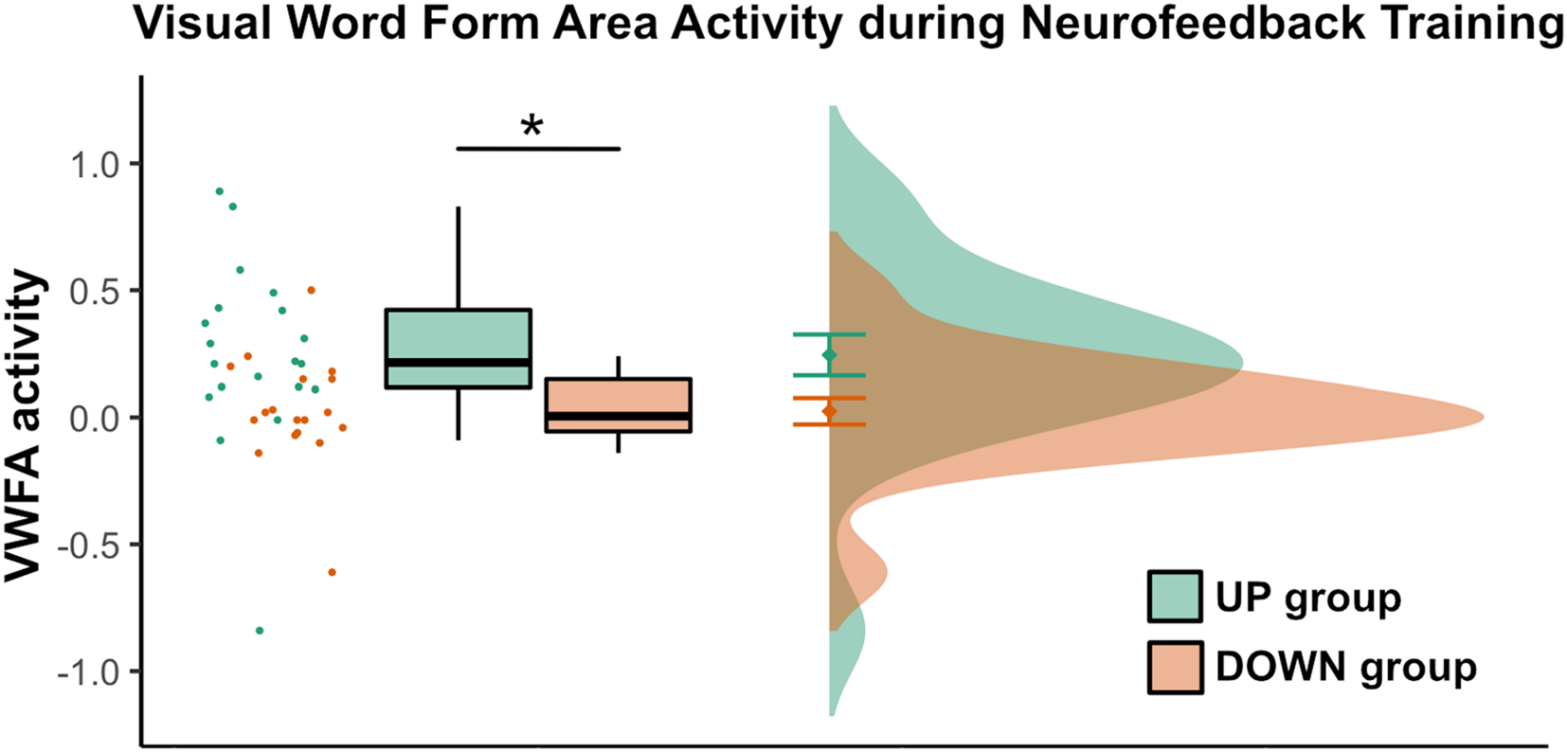
Visual word form area activation during neurofeedback training. We observed a significant difference in VWFA activation during neurofeedback training (mean activation over all six neurofeedback runs) between the UP and DOWN group. Mean values and error bars (depicting one standard error) are displayed on the right side of the boxplots.

For each subject, we extracted mean brain activation within their individual VWFA mask trained during NF. This was done with non-normalized data to replicate activation during online NF training. A mixed ANOVA with factors group (UP, DOWN) and run (1–6) revealed no significant interaction (F(3.10,108.34) = 0.78, p = 0.51). Significant main effects were found for the factors group (F(1,35) = 5.87, p = 0.02) and run (F(3.10,108.34) = 3.24, p = 0.02), indicating stronger activation in the UP group than the DOWN group and differences in activation between runs (see Fig. 4). All ANOVAs were performed using a covariate for VWFA activation during the first functional VWFA localizer run to account for different VWFA baseline activation levels. When excluding this covariate the results remained similar (see Supplemental Material Sect. 4). Further, we did not find any significant difference between the two groups when comparing the absolute values of the difference between the VWFA activation during the first and the second NF run to investigate whether one group would be learning significantly faster than the other group (t(36) = 0.33, p = 0.74). An overview of used mental strategies during NF training is given in the supplemental material.

### Activation in the visual word form area during no-feedback runs before and after neurofeedback training

**Figure 5:**
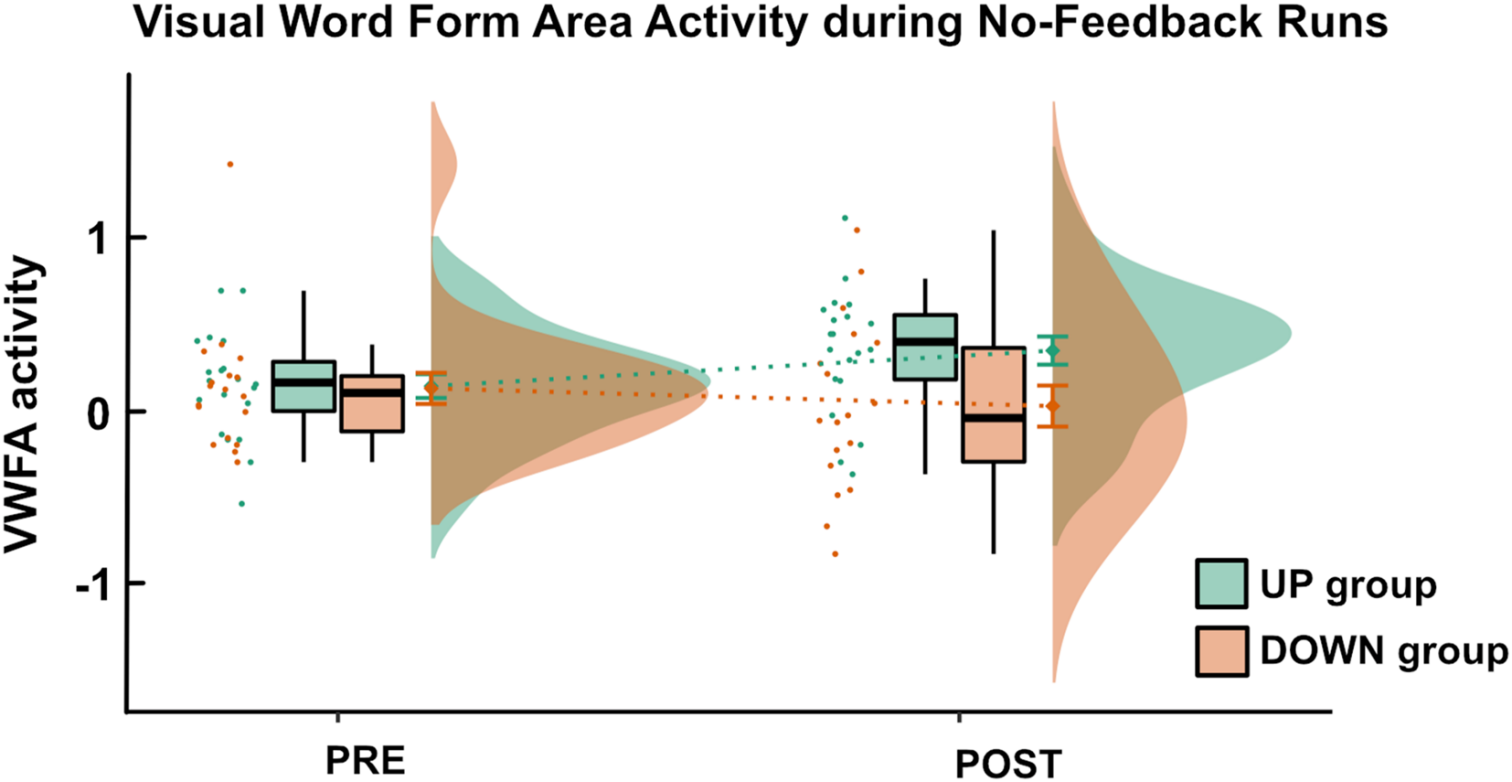
Visual word form area activation during no-feedback runs before and after neurofeedback training. We observed a significant interaction effect between the factors run (PRE, POST) and group (UP, DOWN). Before neurofeedback training, the two groups did not differ significantly, but after neurofeedback training, the UP group demonstrated higher VWFA activation during the no-feedback run than the DOWN group. Mean values and error bars (depicting one standard error) are displayed on the right side of the boxplots.

Similarly to the NF analysis, we extracted VWFA activation during no-feedback runs from each subject’s individual NF target region. A mixed ANOVA with factors group (UP, DOWN) and run (PRE, POST) showed a significant interaction between these two factors (F(1,35) = 5.21, p = 0.03; Fig. 5). Again, baseline VWFA activation during the localizer was used as a covariate (see Supplemental Material Sect. 4 for results without the covariate). Post-hoc tests showed no significant difference between the groups before NF training (F(35) = 0.05, p = 0.83), but significantly higher VWFA activation in the UP group as compared to the DOWN group after NF training (F(35) = 5.32, p = 0.03). Further, they showed a significant increase in activation between the PRE and POST timepoint for the UP group (F(35) = 4.54, p = 0.04), however, no significant change for the DOWN group (F(35) = 1.33, p = 0.26).

### Activation in the visual word form area during the functional VWFA localizer runs before and after neurofeedback training

**Figure 6:**
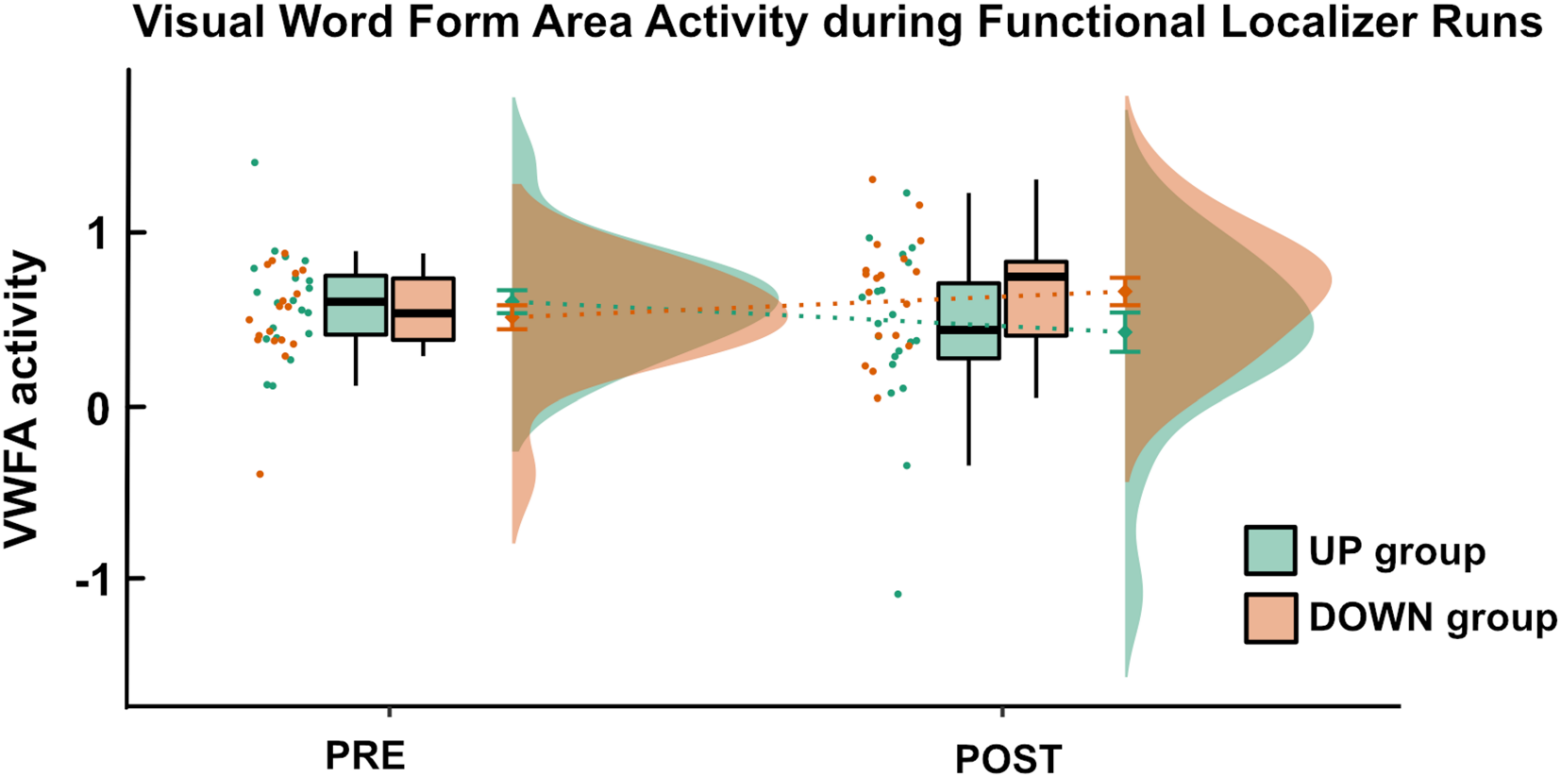
Visual word form area activation during functional VWFA localizer runs before and after neurofeedback training. We found a significant interaction effect between the factors run (PRE, POST) and group (UP, DOWN) for the functional localizer runs. The two groups did not differ significantly in VWFA activation before or after training, but the UP group demonstrated a trend towards a decrease in activation over time while the DOWN group did not show a significant change in VWFA activation over time.

Finally, we also extracted VWFA activation during the functional VWFA localizer runs and compared it before and after NF training. Here, we found a significant interaction between the factors group (UP, DOWN) and run (PRE, POST) (F(1,36) = 6.81, p = 0.01). This interaction showed slightly higher values for the UP than the DOWN group before NF training and lower values for the UP than the DOWN group after NF training (Fig. 6). Post-hoc tests did not show significant group differences between the UP and DOWN group before (F(36) = 0.88, p = 0.36) or after (F(36) = 2.77, p = 0.11) NF training. However, they showed a significant decrease in VWFA activation for the UP group (F(36) = 4.22, p = 0.047). They did not show a significant increase in VWFA activation for the DOWN group (F(36) = 2.72, p = 0.11).

### Comparison between behavioral reading measures before and after neurofeedback training

For completeness, we also investigated the potential effects of time and/or neurofeedback training on reading skills by performing mixed ANOVAs with factors time (pre, post) and group (UP, DOWN). We did not find significant interaction effects for reading fluency of words (F(1,34) = 0.39, p = 0.54) or pseudowords (F(1,34) = 0.13, p = 0.72), reading comprehension (F(1,38) = 0.04, p = 0.85), reading speed (F(1,38) = 0.17, p = 0.68), and reading accuracy (F(1,38) = 2.29, p = 0.14). However, most measures demonstrated significant improvements over time and we observed a significant main effect of time for reading fluency of words (F(1,34) = 22.61, p < 0.001) and pseudowords (F(1,34) = 20.06, p < 0.001), reading comprehension (F(1,38) = 18.85, p < 0.001), and reading speed (F(1,38) = 35.54, p < 0.001). Reading accuracy (F(1,38) = 0.01, p = 0.94) did not show a significant main effect of time. No significant main effect of group was observed for any of the reading measures (see Supplementary Material Sect. 2 for more details).

## Discussion

Here, for the first time, we investigated the feasibility of regulating brain activation within the VWFA using rtfMRI NF. For the NF runs, our results showed significantly stronger VWFA activation in the participant group who had to upregulate their VWFA as compared to the participant group who received instructions to downregulate their VWFA. Moreover, we observed significantly higher VWFA activation in the UP than in the DOWN group in the no-feedback transfer run after NF training. This difference was driven by a significant increase in VWFA activation in the UP group, while the DOWN group did not decrease their VWFA activation significantly. These results indicate that upregulation of the VWFA using mental imagery is indeed feasible.

### Self-regulation of the VWFA

Our NF results clearly demonstrated a significant difference in VWFA activation during NF runs between the UP and DOWN groups. Importantly, the two groups did not show any significant differences in demographic, cognitive, or reading-related measures. This is in line with previous findings of NF studies observing successful self-regulation in the majority of cases (see^52^ for an estimate of NF success in previous studies). However, the two groups differed in their received instructions and corresponding mental imagery.

To investigate the influence of mental imagery performance alone on VWFA activation, we included two no-feedback runs before and after NF training where participants performed the same mental strategies as during NF runs, but no feedback was provided. Importantly, the two groups did not show a significant difference in VWFA activation levels during the no-feedback run prior to NF, but a pronounced difference after NF training. This result nicely shows that, before participants undergo NF training, mental imagery alone seems not sufficient to actively control one’s own VWFA activation.

Consequently, self-regulation of the VWFA can also be performed in the absence of any feedback presentation, but only once this regulation has been practiced, suggesting a successful transfer effect. Importantly, this finding strengthens the potential of VWFA regulation as a therapeutic intervention as it demonstrates that active control of ongoing VWFA activation after NF training and a transfer to everyday life may be possible. This is also in line with other NF studies targeting regions such as the amygdala or the ventral tegmental area that showed successful self-regulation of their respective target areas during no-feedback runs after the NF intervention^27,37^.

Finally, when comparing VWFA activation during the no-feedback runs before and after NF training for each group independently, we found a significant increase for the UP group, but no change in the DOWN group. Potential floor effects might explain this finding as the strategy of the DOWN group was to think of anything but reading which could already have been successful prior to NF training making it difficult to further downregulate. The UP group, in contrast, most likely had more potential to further improve their mental strategies and to, thus, increase their own VWFA activation.

In conclusion, our findings of the no-feedback runs indicate that upregulation of one’s own VWFA activation levels is indeed feasible and that, once this upregulation is learned during NF runs, it can also be performed without the presence of feedback on ongoing VWFA activation levels.

### NF-upregulation vs downregulation training of the VWFA induces activation across the reading network

When comparing the UP to the DOWN group for the regulation versus baseline contrast, we found stronger activation in a range of key regions of the brain’s reading network, including the left STG, left IFG, and left PCG^8^ and using a more lenient initial threshold of 0.005 also the VWFA and the left Supramarginal Gyrus (see Table S6 and Figure S8 in the Supplemental Material). In specific, the cluster in the left STG suggests that participants also engaged in mental articulation by mentally pronouncing the words and letters they visualized. The left STG is associated with both speech perception and speech production^53–55^ and, in the context of reading, has been identified to be involved in the integration of letters and speech sounds^56^. Second, we found a significant cluster in the left IFG which is associated with both phonological and semantic processing and has been found to be active during phonological and semantic verbal fluency tasks^57^. This finding also indicates that participants not only visualized the shape of words and letters but also involved phonological and semantic aspects in their mental imagery. Further, we also observed significant activation in the left PCG, a brain region associated with articulatory movements^58^ and coordination of speech articulation^59^. This indicates that some participants may have covertly articulated the imagined letters, words, and sentences during their mental imagery task.

Taken together, our findings show that upregulation of the VWFA leads to widespread activation increases across the whole reading network as compared to downregulation. These activation increases in additional reading-related brain regions might further enhance potential NF-driven improvements in reading performance as many of these other regions, also, have been found to show hypoactivation in individuals with poor reading skills as compared to those with typical reading skills^16,60,61^.

### VWFA regulation did not influence reading skills in proficient readers

We did not find a significant difference in improvements in reading performance between the UP and the DOWN group. In fact, both groups showed significant improvements in their SLRT-II reading fluency scores for words and pseudowords and their LGVT scores for reading comprehension and reading speed. Only reading accuracy did not improve after NF, neither in the UP nor in the DOWN group. The improvement in reading scores in both groups can most probably be explained by practice effects as the same (SLRT-II) or very similar versions of the tests (LGVT) were performed only a few hours after completion of the first tests^62^. The improvement in speed, but not accuracy, indicates that participants tried to be faster in the tests after the NF intervention, a strategy that particularly impacts the performance of the reading fluency test.

Finally, the lack of difference in improvement between the two groups is most likely due to the fact that our participants were adults with typical reading skills who did not demonstrate any reading deficiencies. Indeed, it is rather unlikely that experienced adults without reading impairments would significantly change their reading speed or accuracy solely due to a single session (~ 30 min) of six short instances of VWFA activation upregulation training. Such interventions may be more meaningful in beginning readers or individuals with reading impairments. This is also in line with neuromodulation interventions using transcranial direct current stimulation (tDCS) which showed specific effectiveness in individuals with reading impairments with regards to improvements in reading performance, but not in typical readers^63,64^. For instance, Marchesotti and colleagues demonstrated significant improvements in phonological processing and reading accuracy after a tDCS intervention of 20 min, but even slightly disturbing effects on individuals without reading impairments^64^. Consequently, future NF intervention studies targeting the VWFA or other parts of the reading network should either investigate individuals with poor reading skills, beginning readers such as children in the progress of learning to read, or adults learning a new script.

Further, it might also be beneficial to perform NF training over a longer time period to strengthen potential behavioral effects and to benefit from sleep consolidation^65^. In addition, recent studies have shown that the positive effect of NF training on clinical and behavioral measures increases over time^66^, most likely due to the fact that, once relevant mental strategies have been learned, participants are able to apply them in their everyday life. As we, also, observed in our study that participants were able to voluntarily increase their VWFA activation during a no-feedback run after NF training, we can assume that these participants would be able to apply their mental strategies over a longer time course outside the MR scanner as well. Consequently, future studies should try to integrate follow-up sessions several days or weeks after the neurofeedback intervention.

### Sustained upregulation of the VWFA led to reduced responsivity to words during passive word viewing

We observed a significant interaction effect between the factors run (PRE, POST) and group (UP, DOWN) for VWFA activation during the localizer runs. Interestingly, this interaction pointed in the opposite direction than expected, namely an increase in VWFA activation from the PRE to the POST run in the DOWN group and a decrease in the UP group. Indeed, post-hoc tests demonstrated a significant decrease in VWFA activation in the UP group but no significant change in VWFA activation in the DOWN group. In comparison, VWFA activation during no-feedback runs increased in the UP group and remained on a similar level in the DOWN group. This indicates that NF training enabled participants to actively upregulate their VWFA using mental imagery, but that upregulation would not automatically result in higher VWFA responsiveness during a passive viewing task after the regulation.

One explanation for this finding might be neural adaptation. Previous studies have shown that the VWFA shows neural adaptation to repeated reading of words, i.e. decreased VWFA activation after a repeated reading task^67,68^. Consequently, the consistent activation of the VWFA using reading-related mental imagery in the UP group might have led to decreased responsiveness during passive viewing. Whether or not the participants in the UP group tried to rehearse and mentally read the words that have been presented during the functional VWFA localizer task during their NF regulation blocks is unfortunately not known. Such rehearsal could however explain to some extent the decrease in activation due to repetition and neural suppression effects. In contrast, the DOWN group did not engage their VWFA during mental imagery and, consequently, likely did not influence VWFA responsiveness to orthographic stimuli.

### Limitations

Adults with typical reading skills as participants allowed for investigating the feasibility of self-regulation of the VWFA in this study. Whether or not our results are generalizable to other participant groups and especially to specific patient groups would need to be examined in future studies. In particular, we cannot conclude whether self-regulation of the VWFA is also feasible in people with dyslexia or in children or adults who are in the process of learning to read. Therefore, in future studies, additional participant groups should be included to investigate the feasibility of VWFA regulation in patient populations or during reading development. Further, our adults did not show any deficits in reading performance, making it less likely to achieve considerable improvements in reading performance that are measurable as a result of successful VWFA upregulation.

Second, all reading tasks and MRI tasks were performed within one single session. Consequently, tiredness and attention might have affected task conduction at the end of the session more than at the beginning of the session. Ideally, tasks should be performed before and after NF training at similar fatigue levels. In addition, performing post-training tests on a different day than NF training might allow for sleep consolidation^65^ which could further improve the effects of NF training. On the other hand, a recent machine learning mega-analysis investigating factors that influence the success of NF studies found no overall beneficial effects of conducting NF studies over several days as compared to just one day^52^.

Third, we could not fully exclude potential baseline effects. While OpenNFT did not take baseline activation into account when calculating the feedback signal, differences in baseline activation between the two groups might still have affected the regulation versus baseline contrasts in the two no-feedback runs. Further, baseline differences might have still effected offline analyses of the NF runs which were also based on regulation versus baseline contrasts.

### Outlook

Here, we demonstrated the feasibility of upregulation of the VWFA using rtfMRI NF. As a next step, further studies are needed to investigate whether successful NF training also leads to improvements in reading skills in specific participant groups with poor reading skills or participants who are in the process of learning to read to avoid ceiling effects. As we observed large parts of the reading network to be activated during upregulation as compared to downregulation, the inclusion of additional regions of the reading network might also be possible to boost aspects of the reading process other than visual processing. Finally, future applications of VWFA regulation might also make use of cheaper, more flexible methods such as electroencephalography (EEG), for instance by generating an EEG fingerprint of the fMRI-based VWFA signal and training the fingerprint signal^69,70^.

## Supplementary Information

Supplementary Information.

## Data Availability

All code used for MRI data analyses can be found on our Open Science Framework repository: https://osf.io/4qhdb/. The repository also includes exam cards with detailed information on MR parameters and the literature-based mask of the Visual Word Form Area. Data will be made available upon written request to the corresponding author. A formal data sharing agreement will be required.
