# Supplementary material for: Self-regulation of visual word form area activation with real-time fMRI neurofeedback
Source: Sci Rep. 2023 Jun 6;13:9195. doi: 10.1038/s41598-023-35932-9 (PMC10244457; doi:10.1038/s41598-023-35932-9)
Supplement: Supplementary file 1 — Supplementary Information. [file 41598_2023_35932_MOESM1_ESM.pdf]

Supplementary Material

1. Literature-based Visual Word Form Area Mask

The literature-based VWFA mask was created by defining spheres with different radii around the reported activation peaks of the articles on VWFA listed below using the Marsbar toolbox for SPM (https://marsbar-toolbox.github.io/). These spherical ROIs were then combined to form a joint VWFA mask and used to identify individual ROIs for neurofeedback training and for the analyses of training effects and activation in the functional VWFA localizer task.

**Literature**	**Coordinates (x,y,z in MNI space)**	**Right hemisphere correlate**	**Radius**
Impact of literacy on the functional connectivity of vision and language related networks (López-Barroso et al., 2020)	-44, -50, -14	44, -50, -14	8 mm
Brain sensitivity to print emerges when children learn letter–speech sound correspondences (Brem et al., 2010)	-48, −66, −14;	48, −66, −14;	6 mm
Variability in Location Impacts Orthographic Selectivity in the "Visual Word Form Area" (Glezer & Riesenhuber, 2013)	-45, -56, -16	45, -56, -16	4 mm
Lateralized task shift effects in Broca's and Wernicke's regions and in visual word form area are selective for conceptual content and reflect trial history (Wallentin, Michaelsen, Rynne, & Nielsen, 2014)	-43, -54, -12	43, -54, -12	10 mm
The Putative Visual Word Form Area Is Functionally Connected to the Dorsal Attention Network (Vogel, Miezin, Petersen, & Schlaggar, 2012)	-45, -57, -12	45, -57, -12	4 mm

The VWFA Is the Home of Orthographic Learning When Houses Are Used as Letters (Martin et al., 2019)	-34, -55, -13	34, -55, -13	6 mm
Converging evidence for functional and structural segregation within the left ventral occipitotemporal cortex in reading (Lerma-Usabiaga, Carreiras, & Paz-Alonso, 2018)	-42, -58, -10	42, -58, -10	6 mm

Table S1. Coordinates and radii of the spheres chosen based on literature. A general visual word form area mask was created based on previous literature.

References

Brem, S., Bach, S., Kucian, K., Guttorm, T. K., Martin, E., Lyytinen, H., … Richardson, U. (2010). Brain sensitivity to print emerges when children learn letter-speech sound correspondences. *Proceedings of the National Academy of Sciences of the United States of America*, *107*(17), 7939–7944. https://doi.org/10.1073/pnas.0904402107

Glezer, L. S., & Riesenhuber, M. (2013). Individual variability in location impacts orthographic selectivity in the "visual word form area." *Journal of Neuroscience*, *33*(27), 11221–11226. https://doi.org/10.1523/JNEUROSCI.5002-12.2013

Lerma-Usabiaga, G., Carreiras, M., & Paz-Alonso, P. M. (2018). Converging evidence for functional and structural segregation within the left ventral occipitotemporal cortex in reading. *Proceedings of the National Academy of Sciences of the United States of America*, *115*(42), E9981–E9990. https://doi.org/10.1073/pnas.1803003115

López-Barroso, D., Thiebaut de Schotten, M., Morais, J., Kolinsky, R., Braga, L. W., Guerreiro-Tauil, A., … Cohen, L. (2020). Impact of literacy on the functional connectivity of vision and language related networks. *NeuroImage*, *213*(March). https://doi.org/10.1016/j.neuroimage.2020.116722

Martin, L., Durisko, C., Moore, M. W., Coutanche, M. N., Chen, D., & Fiez, J. A. (2019). The VWFA is the home of orthographic learning when houses are used as letters. *ENeuro*, *6*(1), 1–13. https://doi.org/10.1523/ENEURO.0425-17.2019

Vogel, A. C., Miezin, F. M., Petersen, S. E., & Schlaggar, B. L. (2012). The putative visual word form area is functionally connected to the dorsal attention network. *Cerebral Cortex*, *22*(3), 537–549. https://doi.org/10.1093/cercor/bhr100

Wallentin, M., Michaelsen, J. L. D., Rynne, I., & Nielsen, R. H. (2014). Lateralized task shift effects in Broca's and Wernicke's regions and in visual word form area are selective for conceptual content and reflect trial history. *NeuroImage*, *101*, 276–288. https://doi.org/10.1016/j.neuroimage.2014.07.012

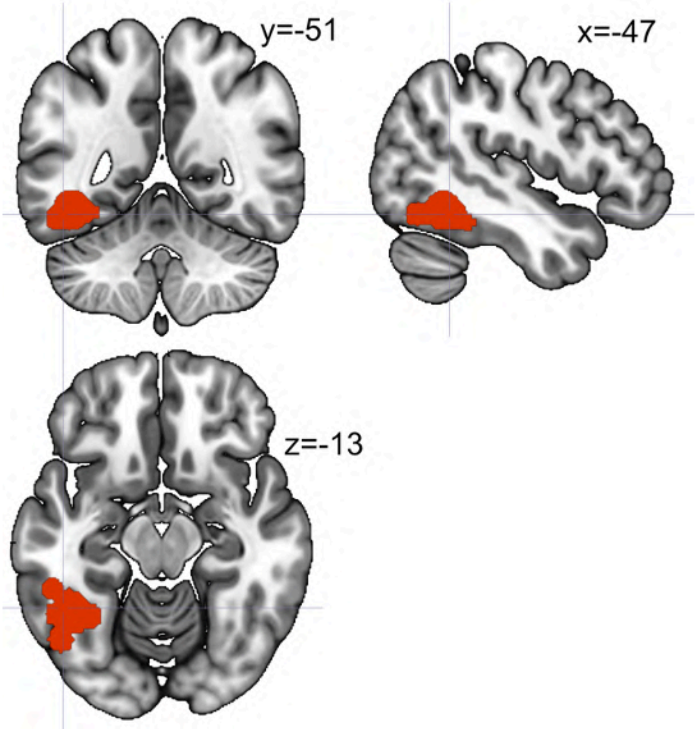

Figure S1. Literature-based mask of the Visual Word Form Area. The literature-based Visual Word Form Area (VWFA) mask was created by combining spheres of varying radii with coordinates and radii taken from the literature on the VWFA (See Table S1).

2. Behavioral results

No main effect of group (UP, DOWN) was found for any of the reading measures

No significant main effect of group was found for reading fluency of pseudowords ($F(1,34)=2.55$, $p=0.12$) or words ($F(1,34)=1.95$, $p=0.17$), or for reading accuracy ($F(1,38)=0.45$, $p=0.51$), reading speed ($F(1,38)=0.77$, $p=0.39$), and reading comprehension ($F(1,38)=0.25$, $p=0.62$).

No correlation between changes in reading measures and VWFA engagement during neurofeedback training

Changes in reading fluency were not significantly correlated to mean VWFA activation during neurofeedback training, neither for words ($r(35)=0.08$, $p=0.65$), nor for pseudowords ($r(35)=0.16$, $p=0.33$) (all correlations were corrected for group assignment). Further, we did not observe significant correlations between VWFA activation during neurofeedback training and changes in reading comprehension ($r(29)=0.16$, $p=0.40$), reading speed ($r(29)=0.20$, $p=0.29$), or reading accuracy ($r(29)-0.12$, $p=0.54$).

3. Functional VWFA localizer results before neurofeedback training (PRE)

Brain regions engaged by the functional VWFA localizer task in the UP group (N=20)

**cluster**		**peak**					**anatomy**
p(FWE-corr)	equivk	p(FWE-corr)	T	x	y	z {mm}	
<0.001	4022	0	14.58	-34	-92	-8	left inferior occipital gyrus, left fusiform gyrus, including VWFA
		0	12.21	-44	-66	-18	
		0.004	8.51	-34	-46	-20	
<0.001	1904	0	11.14	36	-86	-8	right inferior occipital gyrus
		0	10.69	32	-92	-2	
		0.103	6.33	4	-74	-32	
<0.001	4950	0.002	8.82	-42	8	26	left precentral gyrus
		0.019	7.43	-52	2	28	
		0.033	7.06	-50	20	16	
<0.001	1527	0.23	5.79	-48	-46	44	left supramarginal gyrus, left angular gyrus
		0.284	5.65	-28	-58	48	
		0.385	5.42	-28	-68	56	
0.004	343	0.255	5.72	54	-10	50	right precentral gyrus
		0.365	5.46	38	-12	64	
		0.581	5.07	38	-28	66	
0.015	263	0.266	5.69	-28	-18	-6	left putamen
		0.292	5.63	-38	-18	-20	
		0.749	4.79	-28	-14	-18	
0.001	487	0.396	5.4	-2	2	72	supplementary motor cortex
		0.709	4.86	-8	-2	60	
		0.826	4.65	-4	26	46	
0.026	231	0.497	5.21	50	-32	-2	right middle temporal gyrus
		1	3.6	52	-38	-10	

Table S2. Whole brain activation during the functional VWFA localizer in the UP group pre training. Initial threshold $p < 0.001$ uncorrected.

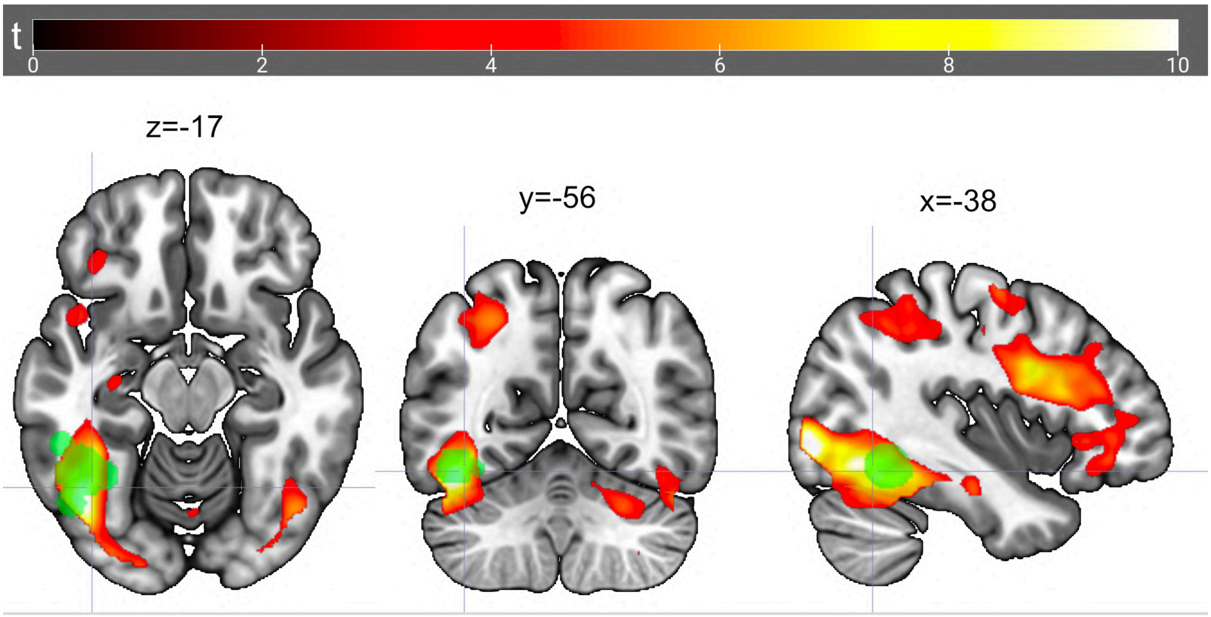

Figure S2. Whole brain activation during the functional localizer in the UP group pre training. The Visual Word Form Area mask is highlighted in green. Initial threshold p < 0.001 uncorrected.

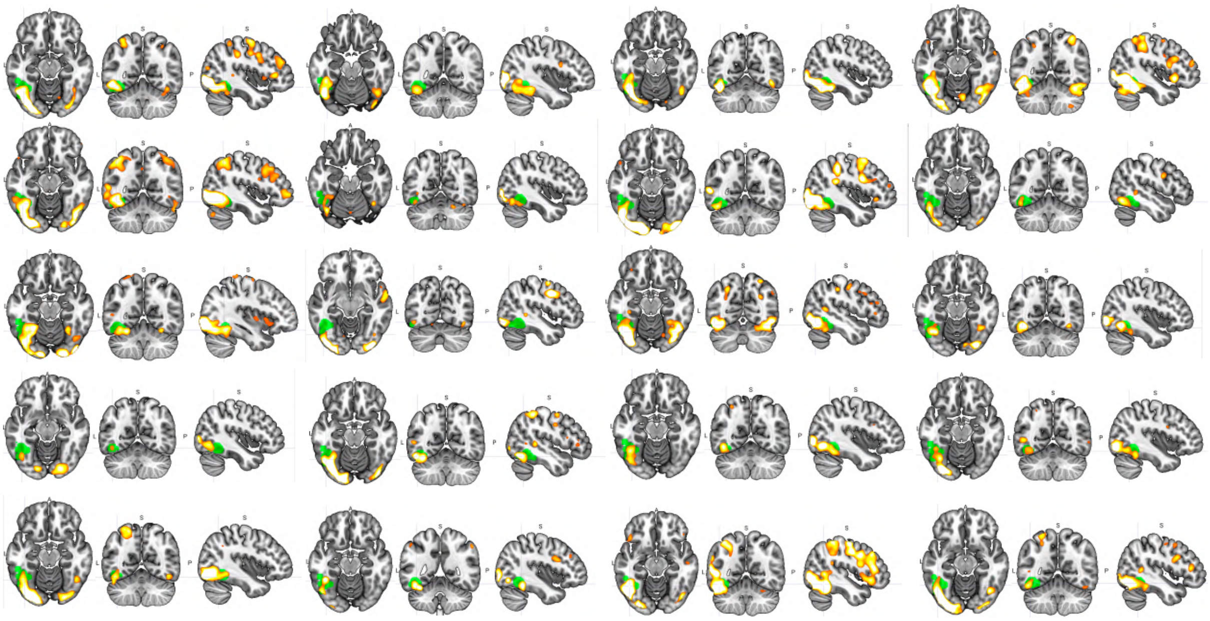

Figure S3. Individual whole brain activation during the functional VWFA localizer for individuals of the UP group pre training. The sagittal view shows the left hemisphere. All figures were created with an initial threshold of p < 0.001 uncorrected.

Brain regions engaged by the functional VWFA localizer task in the DOWN group (N=20) pre training

**cluster**		**peak**					**anatomy**
p(FWE -corr)	equivk	p(FWE -corr)	T	x	y	z {mm}	
<0.001	2696	0	15.22	-44	-64	-18	left fusiform gyrus, including VWFA, left inferior occipital gyrus
		0	12.2	-34	-88	-8	
		0	10.34	-38	-46	-22	
<0.001	707	0.002	9.39	-30	-10	-14	left amygdala, left putamen
		0.635	5.11	-26	-2	10	
		0.692	5.01	-38	-12	-28	
0.011	270	0.011	8.08	28	-90	-4	right inferior occipital gyrus
<0.001	2537	0.016	7.8	-36	34	18	left middle frontal gyrus
		0.06	6.89	-44	2	28	
		0.202	6.05	-28	4	24	
<0.001	635	0.354	5.63	-50	-40	38	left supramarginal gyrus
		0.532	5.29	-52	-38	46	
		0.927	4.51	-44	-44	60	
0.012	262	0.426	5.48	26	-70	-44	right cerebellum
		0.643	5.09	18	-76	-46	
		0.727	4.95	24	-70	-52	
0.046	188	0.529	5.29	-8	4	68	supplementary motor cortex
		0.998	3.94	4	2	66	

Table S3. Whole brain activation during the functional VWFA localizer in the DOWN group pre training. Initial threshold $p < 0.001$ uncorrected.

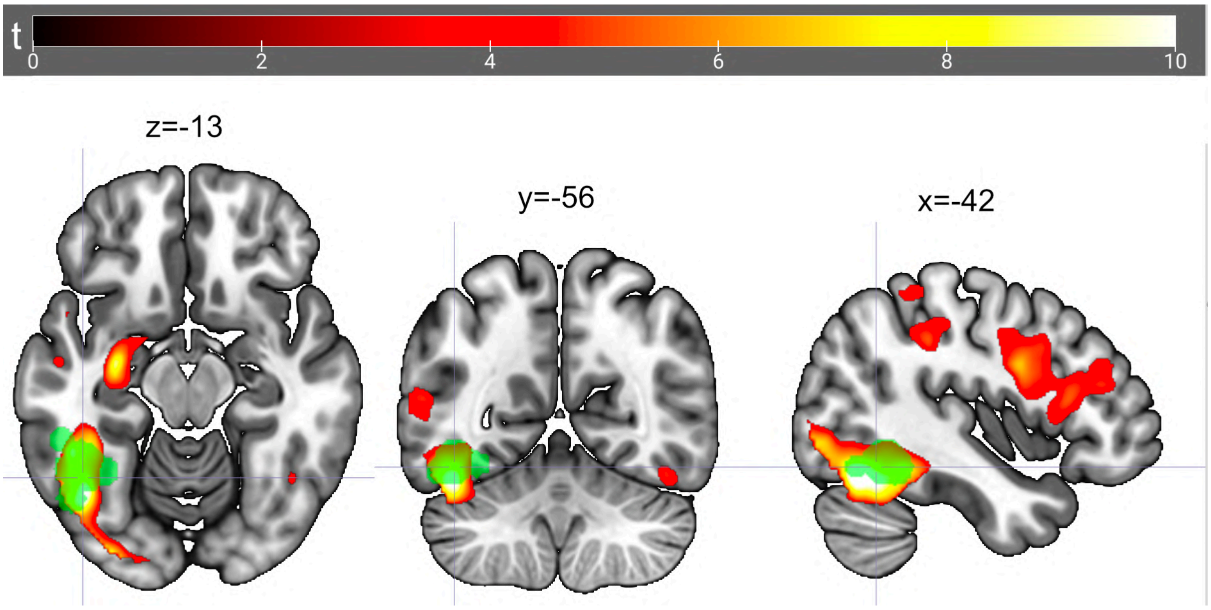

Figure S4. Whole brain activation during the functional VWFA localizer in the DOWN group pre training. The Visual Word Form Area mask is highlighted in green. Initial threshold p < 0.001 uncorrected.

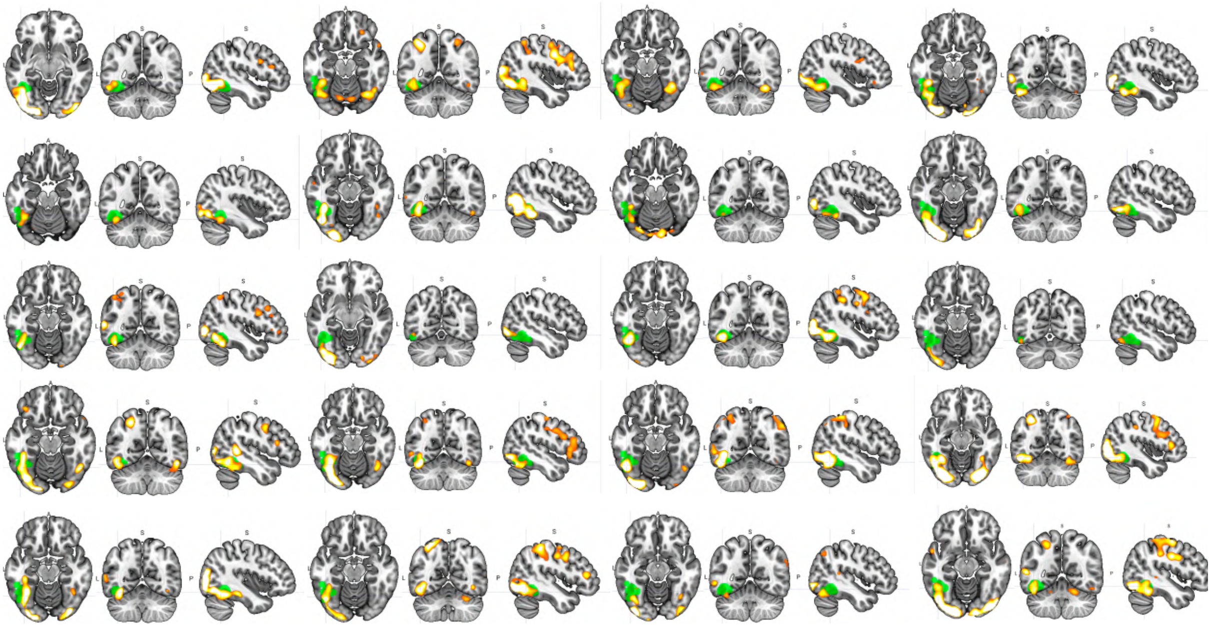

Figure S5. Individual whole brain activation during the functional VWFA localizer for individuals of the DOWN group pre training. The sagittal view shows the left hemisphere. All figures were created with an initial threshold of p < 0.001 uncorrected.

Whole brain group differences between the UP and DOWN group during the functional localizer task prior to neurofeedback training

We did not observe any significant (cluster corrected) clusters when comparing functional localizer activation between the two groups.

4. Neurofeedback results

Whole brain activation for the regulation versus baseline contrast in the UP group

The UP group who was instructed to upregulate their own VWFA activation demonstrated significant clusters across the whole reading network when contrasting regulation to baseline blocks. In specific, we observed significant activation within the bilateral ventral occipital cortex including the VWFA, the bilateral inferior frontal gyrus and bilateral precentral gyrus, the left posterior STG, and the bilateral supramarginal gyrus. In addition, we found significant clusters covering the bilateral anterior insula, the bilateral pallidum, caudate, and ventral tegmental area, and the bilateral supplementary motor area and superior parietal lobule (see Table S4 for detailed coordinates).

**cluster**		**peak**		**coordinates**			**anatomy**
p(FWE -corr)	equivk	p(FWE -corr)	T	x	y	z {mm}	
<0.001	9030	0	12.65	34	26	-2	right anterior insula<br>right IFG<br>including right precentral gyrus
		0	10.39	50	12	18	
		0	10.16	46	14	26	
<0.001	7111	0	10.08	-40	24	-4	left IFG<br>including left precentral gyrus<br>left anterior insula
		0	10.07	-38	32	-2	
		0.001	9.37	-30	24	-6	
<0.001	6506	0.001	9.3	40	-72	18	occipital gyrus<br>right fusiform gyrus
		0.004	8.3	46	-66	10	
		0.006	8.08	44	-64	-10	
<0.001	1889	0.011	7.69	-8	20	44	left supplementary motor area
		0.014	7.56	-8	8	58	
		0.014	7.52	6	20	42	
<0.001	1195	0.012	7.63	-24	-54	44	left superior parietal lobule, left supramarginal gyrus
		0.107	6.22	-24	-66	48	
		0.376	5.35	-44	-40	40	
0.003	487	0.06	6.59	-44	-66	-8	left ventral occipito-temporal cortex (VWFA)
		0.488	5.14	-46	-56	-12	

0.009	387	0.163	5.94	-52	-46	8	left posterior STG

Table S4: Whole brain activation for the regulation versus baseline contrast in the UP group

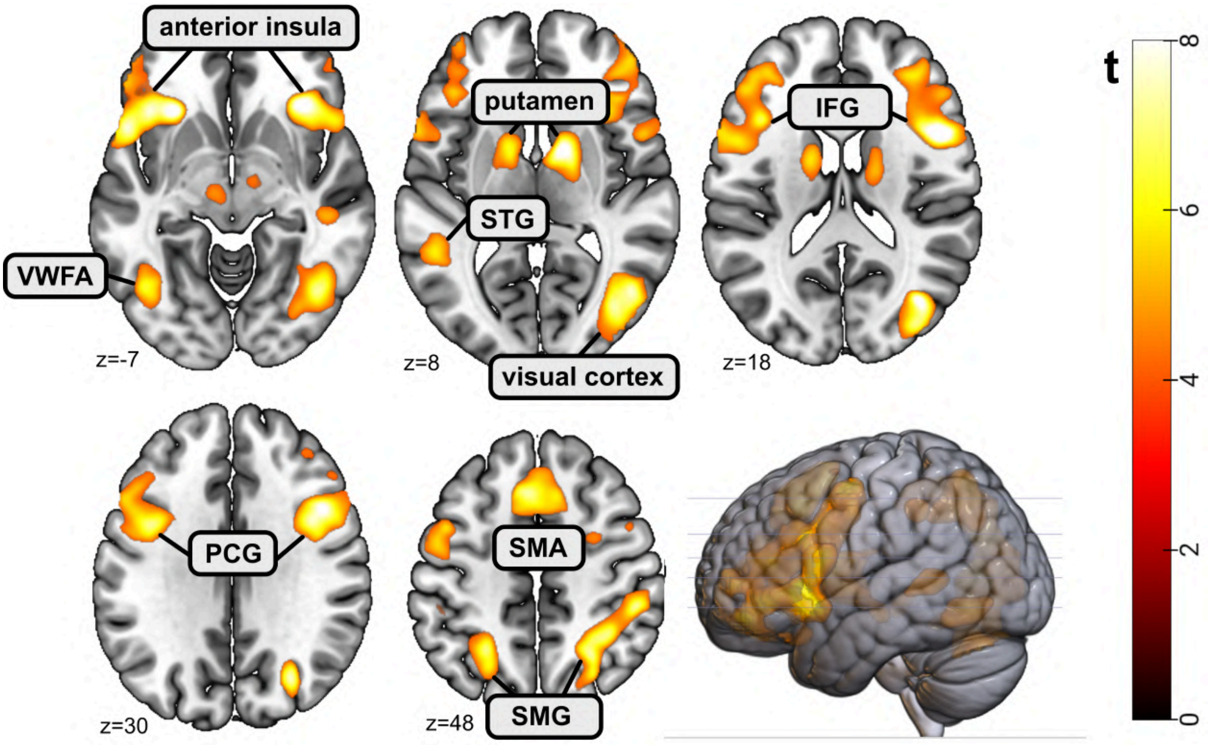

Figure S6: Whole brain activation for the regulation versus baseline contrast in the UP group. Initial threshold p < 0.001 uncorrected. Abbreviations: visual word form area (VWFA), superior temporal gyrus (STG), inferior frontal gyrus (IFG), precentral gyrus (PCG), supplementary motor area (SMA), supramarginal gyrus (SMG).

Whole brain activation for the regulation versus baseline contrast in the DOWN group

In the DOWN group who was instructed to downregulate their VWFA activation we observed significant activation in the right ventral occipital cortex, the right superior parietal lobule, the right supramarginal gyrus, the right precentral gyrus, the right inferior frontal gyrus, the right supplementary motor area, and the bilateral anterior insula (see Table S5 for detailed coordinates).

cluster		peak						
p(FWE -corr)	equivk	p(FWE -corr)	p(FDR -corr)	T	x	y	z {mm}	anatomy
<0.001	4164	0	0.002	11.28	46	10	22	right precentral gyrus right inferior frontal gyrus
		0.009	0.013	8.34	38	38	28	
		0.018	0.017	7.81	30	24	2	
<0.001	4961	0.001	0.004	10.13	46	-68	-10	right inferior occipital gyrus right superior parietal lobule right supramarginal gyrus
		0.004	0.009	8.94	26	-60	46	
		0.005	0.009	8.85	38	-86	18	
0.018	342	0.129	0.079	6.37	6	14	54	right supplementary motor area
		0.335	0.151	5.64	8	28	42	
0.046	266	0.372	0.151	5.55	-28	22	0	left anterior insula

Table S5: Whole brain activation for the regulation versus baseline contrast in the DOWN group

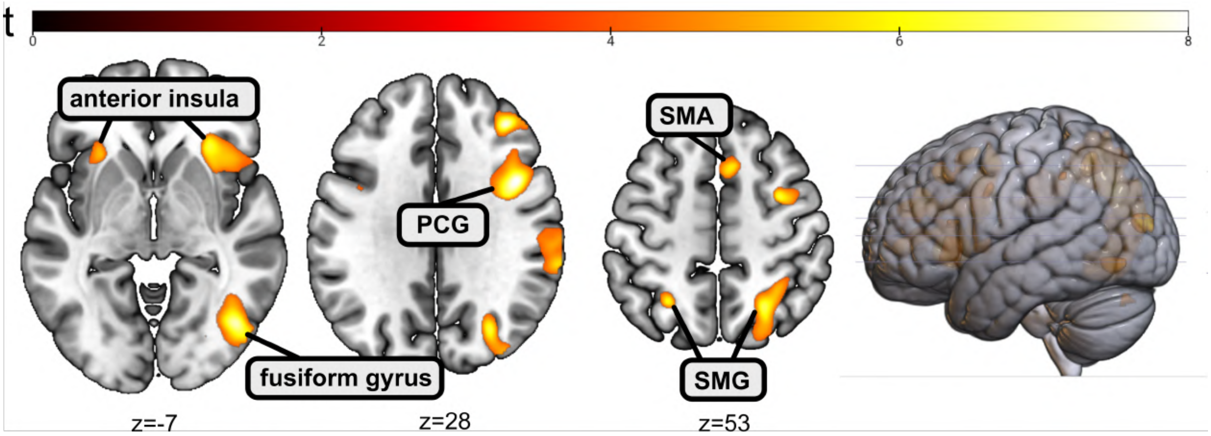

Figure S7: Whole brain activation for the regulation versus baseline contrast in the DOWN group. Initial threshold p < 0.001 uncorrected. Abbreviations: precentral gyrus (PCG), supplementary motor area (SMA), supramarginal gyrus (SMG).

Difference in whole brain activation between the UP and DOWN group for the regulation versus baseline contrast using a liberal initial threshold of 0.005

On a whole brain level, when contrasting the UP to the DOWN group we found significant (initial threshold p<0.005, FWE-corrected p<0.05) clusters across the whole reading network including the left precentral gyrus (PCG), the left IFG, the left supramarginal gyrus, the left STG, and the left vOT including the VWFA. In addition, we observed significant activation within the cuneus the supplementary motor area (SMA), the cerebellum, and the orbitofrontal cortex (OFC) (see Figure S8 and Table S6).

**cluster**		**peak**		**coordinates**			**anatomy**
p(FWE-corr)	equivk	p(FWE-corr)	T	x	y	z {mm}	
<0.001	2295	0.001	6.97	24	-62	-24	cerebellum, including a cluster in the visual word form area
		0.857	3.94	-18	-58	-24	
		0.939	3.77	42	-62	-30	
0.048	726	0.007	6.25	-8	6	60	supplementary motor area
		0.744	4.1	-12	20	40	
		0.997	3.43	-10	30	42	
<0.001	2120	0.026	5.71	-56	-6	40	left precentral gyrus
		0.065	5.35	-48	-8	52	
		0.176	4.93	-56	2	34	
<0.001	2155	0.062	5.37	-42	28	-4	left inferior frontal gyrus, left orbitofrontal cortex
		0.367	4.57	-42	46	-12	
		0.386	4.55	-52	10	-6	
0.048	723	0.072	5.31	-52	-46	8	left superior temporal gyrus
0.001	1586	0.226	4.81	36	52	-14	right orbitofrontal cortex
		0.339	4.61	54	14	-18	
		0.692	4.17	26	18	-24	
0.004	1242	0.586	4.29	18	-86	24	occipital cortex
		0.833	3.98	-8	-98	16	
		0.994	3.48	-8	-88	16	
0.01	1042	0.673	4.19	-28	-38	44	left supramarginal gyrus
		0.771	4.07	-30	-60	56	
		0.826	3.99	-44	-38	40	

Table S6. Overview of clusters demonstrating higher activity in the UP than in the DOWN group for the regulation versus baseline contrast of neurofeedback runs. Initial threshold p<0.005.

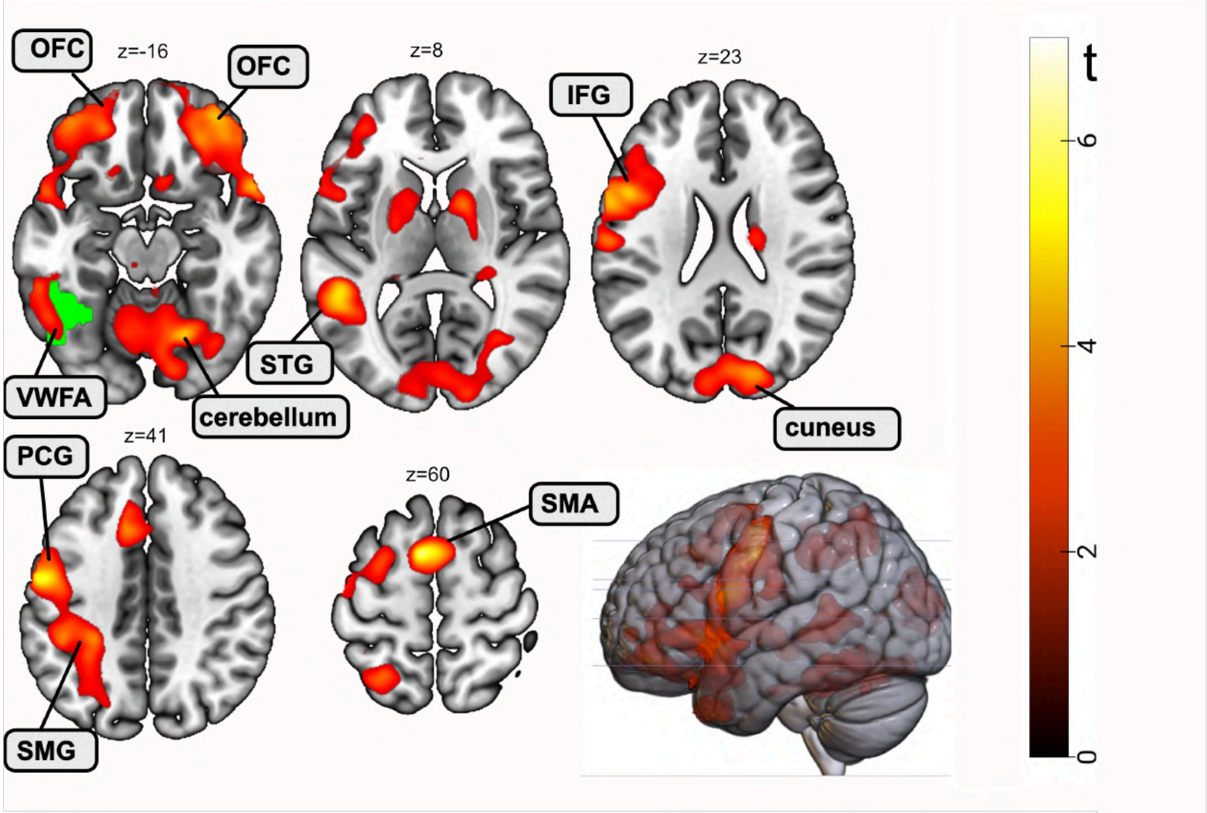

Figure S8: Whole brain activity during neurofeedback runs in the UP group as compared to the DOWN group. When comparing the regulation versus baseline contrast between the two groups, we observed significant (FWE-corrected $p<0.05$) clusters in the visual word form area (VWFA), the left superior temporal gyrus (STG), the left inferior frontal gyrus (IFG), the left precentral gyrus (PCG), the left supramarginal gyrus (SMG), the cerebellum, orbitofrontal cortex (OFC), cuneus, and the supplementary motor area (SMA). The literature-based mask of the VWFA is depicted in green. Initial threshold $p<0.005$

Activation in the Visual Word Form Area during neurofeedback training without a correction for baseline Visual Word Form Area activation levels during the localizer run

A mixed model ANOVA with conditions group (UP, DOWN) and run (1-6) revealed no significant interaction ($F(3.16,113.74)=0.90$, $p=0.45$). A significant main effect was found for both the condition run ($F(3.16,113.74)=0.05$, $p<0.001$), and the condition group ($F(1,36)=5.13$, $p=0.03$).

Activation in the Visual Word Form Area during no-feedback runs without a correction for baseline Visual Word Form Area activation levels during the localizer run

A mixed model ANOVA with conditions group (UP, DOWN) and run (1-6) revealed a trend for an interaction ($F(1,36)=3.77$, $p=0.06$). No significant main effect was found for both the condition run ($F(1,36)=0.40$, $p=0.53$) and the condition group ($F(1,36)=2.79$, $p=0.10$).

Mental strategies used during neurofeedback training

Participants in the UP group were encouraged to use reading-related mental strategies during NF training. As a result, the most used mental strategy was the mental imagery of words, followed by the mental imagery of reading per se. Other reading-related strategies were the mental imagery of writing and the mental imagery of letters. Very few strategies differed from these categories, such as the mental imagery of spelling.

Participants in the DOWN group were asked to use mental strategies unrelated to reading. Here, we observed a larger variety in strategies. The most used mental imagery category was the mental imagery of doing sports, which varied from the mental imagery of hiking to the mental imagery of ball sports. Another popular mental imagery category was thinking of memories and the past or mindfulness strategies.

Lateralization

In this study, left-handed individuals were excluded to increase the power of group analyses with a more homogeneous sample. In fact, 95% of right-handed individuals have been found to show a left-hemispheric dominance of their language network (Springer et al., 1999) while this appears to be the case for only 78% of left-handed and ambidextrous individuals (Szaflarski et al., 2002). However, the individualized nature of the functional localizer task as well as the NF intervention would also allow for NF training of left-handed individuals. The very robust results of the functional localizer task emphasize that it is possible to identify the lateralization as well as location of the VWFA on a single-subject level. This information can then be used to create an individualized NF target for each participant, regardless of the lateralization of this individual's VWFA.

5. References

Springer, J. A., Binder, J. R., Hammeke, T. A., Swanson, S. J., Frost, J. A., Bellgowan, P. S. F., … Mueller, W. M. (1999). Language dominance in neurologically normal and epilepsy subjects. A functional MRI study. *Brain*, *122*(11), 2033–2045. https://doi.org/10.1093/brain/122.11.2033

Szaflarski, J. P., Binder, J. R., Possing, E. T., McKiernan, K. A., Ward, B. D., & Hammeke, T. A. (2002). Language lateralization in left-handed and ambidextrous people: fMRI data. *Neurology*, *59*(2), 238–244. https://doi.org/10.1212/WNL.59.2.238
